# Regulation of proliferation and survival of B-lymphocytes by Ebf1 - implications in leukaemia

**DOI:** 10.1186/1868-7083-5-S1-S4

**Published:** 2013-08-19

**Authors:** Ildikó Győry, Rudolf Grosschedl

**Affiliations:** 1 University of Leicester, Leicester, Leicestershire, LE1 9HN, UK; 2 Max Planck Institute of Immunobiology and Epigenetics, Freiburg, Baden-Württemberg, D-79108, Germany

## 

Ebf1 is an important determinant of the specification of B lymphocytes. However, expression of this transcription factor is not limited to early B lymphopoiesis and constitutive deletion of *Ebf1* results in developmental block and embryonic lethality. To gain insight into additional functions of Ebf1, we inactivated Ebf1 at various stages of differentiation using a Cre-lox-regulated conditional knockout allele.

We found that Ebf1 is required for the proliferation and survival of pro-B cells and peripheral B-cell subsets. In Ebf1-deficient pro-B cells, overexpression of the pro-survival genes Bcl2 or BclXL was not sufficient for cell–survival.

We found a remarkable clonal difference in the cell cycle response of transformed pro-B cells upon induced Ebf1-deletion: one cell type arrested in the G(1) phase and the other type died without arrest. Unexpectedly, the cycling cell-type survived upon c-myb overexpression, in contrast to the other transformed clone type or to primary pro-B cells, showing cell cycle arrest as well. This point to two different Ebf1 regulated cell-cycle-coupled cell survival pathways. As inactivating mutations of the *EBF1* gene are frequent in acute lymphoblastic leukaemia, elucidating the role of Ebf1 in cell cycle and survival could provide novel means of intervention in the growth of leukaemia cells.

